# Acute Cardiovascular Response to Ophthalmic Phenylephrine During Plaque Radiotherapy Insertion

**DOI:** 10.1155/carm/9515014

**Published:** 2026-03-09

**Authors:** Daniel A. Balikov, Nathan L. Scott, Neil H. Masters, Zelia M. Correa

**Affiliations:** ^1^ Bascom Palmer Eye Institute, University of Miami, Miami, Florida, 33136, USA, miami.edu; ^2^ Viterbi Family Department of Ophthalmology, Shiley Eye Institute, University of California San Diego, San Diego, California, 92097, USA, ucsd.edu; ^3^ Moores Cancer Center at University of California San Diego, San Diego, California, 32037, USA; ^4^ Department of Anesthesia, University of Miami, Miami, Florida, 33136, USA, miami.edu; ^5^ Sylvester Comprehensive Cancer Center, University of Miami, Miami, Florida, 33136, USA, miami.edu

**Keywords:** arrhythmia, blood pressure, choroidal melanoma, phenylephrine, plaque radiotherapy

## Abstract

**Purpose:**

To report a case of an acute rise in blood pressure and acquired cardiac arrhythmia resulting from excessive topical phenylephrine exposure intraoperatively during the surgical insertion of a radioactive plaque for choroidal melanoma.

**Methods:**

Observational case report with literature review.

**Observations:**

A 56‐year‐old asymptomatic man with a diagnosis of choroidal melanoma of the right eye underwent routine plaque radiotherapy placement. Due to delayed dilation, additional topical 10% phenylephrine drops were placed on the eye after limited conjunctival peritomy. Shortly thereafter, the patient experienced an acute hypertensive episode with a cardiac arrhythmia and confusion due to the excess phenylephrine used that pooled on the ocular surface. The patient was successfully treated by the anesthesia team and found to have no chronic cardiac conditions. The choroidal melanoma was also successfully treated.

**Conclusion and Importance:**

Additional mydriatic agents may be used with caution in the postincision setting, but should be thoroughly rinsed to prevent adverse cardiac events, particularly phenylephrine. Open communication is critical between the surgical and the anesthesia team prior to the addition of phenylephrine drops.

## 1. Introduction

Appropriate pupil dilation is a key component of several ophthalmic surgeries for both anterior and posterior segments. A visible red reflex during cataract surgery provides the surgeon with excellent lighting and contrast to assess changes to the lens capsule and the cataract, and maximal dilation during retinal detachment repair allows for easier visualization of far peripheral anatomy where causative retinal pathology may hide. Pupillary dilation is achieved essentially by two classes of pharmacologic agents, anticholinergics (e.g., tropicamide) and sympathomimetics (e.g., phenylephrine), that are applied topically in the preoperative setting [1]. Despite the generally safe profile of topical ocular formulations, they do come with risks for adverse reactions of variable intensity if enough medication enters systemic circulation. In the case of phenylephrine, adverse effects include hypertensive episodes and cardiac arrhythmias [2]. Herein, we outline a case of a middle‐aged man undergoing plaque radiotherapy insertion and prognostic transvitreal fine needle aspiration biopsy (FNAB) for choroidal melanoma who experienced acute cardiac adverse effects associated with the inadvertent use of high‐concentration topical phenylephrine.

## 2. Case Presentation

A 56‐year‐old Caucasian man was referred for evaluation of suspected choroidal melanoma associated with an exudative retinal detachment of the right eye after experiencing one month of blurred vision. Ocular history at the time was notable only for presbyopia, normal angles without elevated intraocular pressure. The left eye was normal. Fundus examination of the right eye revealed a large vascularized amelanotic melanocytic choroidal tumor centered at the 8:00 o’clock meridian in the near mid‐periphery measuring 10–10.5 mm in diameter with a superior eruption causing a mushroom shape located superotemporally (Figure 1(a)). Thickness was measured at 8.2 mm on A‐ and B‐scan (Figure 1(b)). In addition, there was a bullous inferior retinal detachment spanning 3:00−9:00, abutting the inferotemporal arcade. Baseline systemic workup revealed a right upper lung nodule not suspected to be of neoplastic origin on chest CT. Because the tumor clinical features were consistent with choroidal melanoma, we recommended treatment using an I‐125 plaque and assessment of tumor prognosis with a prognostic transvitreal FNAB. The pros and cons of this management recommendation were discussed with the patient and his wife, and they agreed to proceed.

FIGURE 1Fundus photography and B‐scan ultrasound imaging. Fundus photo (a) and B‐scan ultrasound (b) imaging at presentation demonstrating an elevated choroidal mass. One year after plaque radiotherapy, the lesion was observed to be regressing (fundus photo (c) and B‐scan ultrasound image (d)), and with resolution of the lesion two years after plaque radiotherapy (fundus photo (e) and B‐scan ultrasound image (f)).

**Figure:**
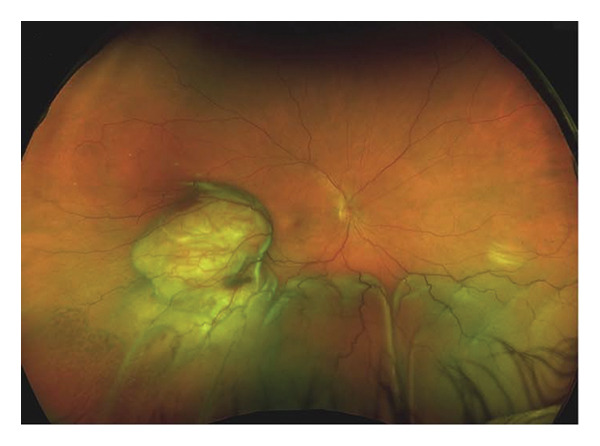
(a)

**Figure:**
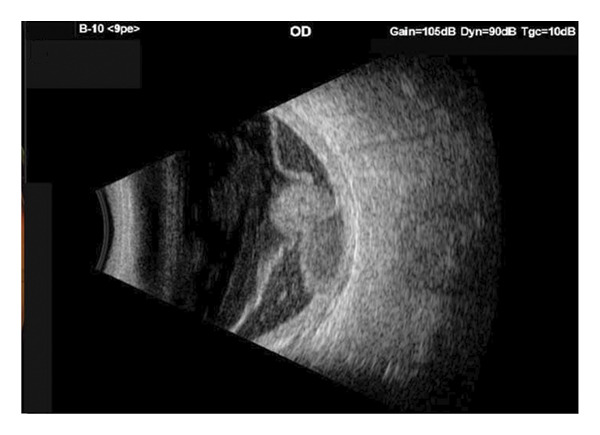
(b)

**Figure:**
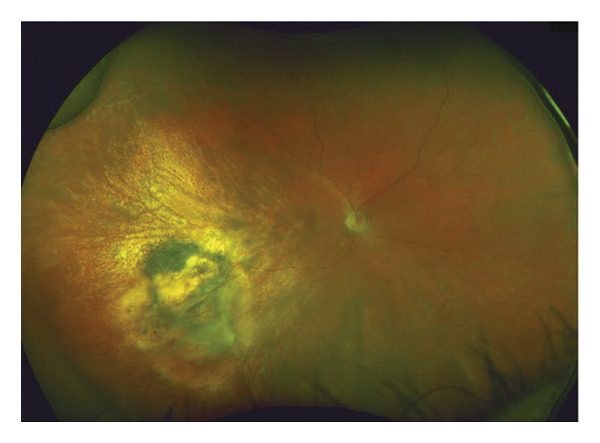
(c)

**Figure:**
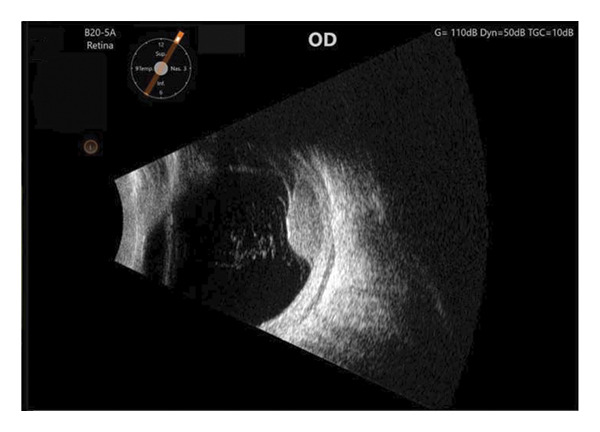
(d)

**Figure:**
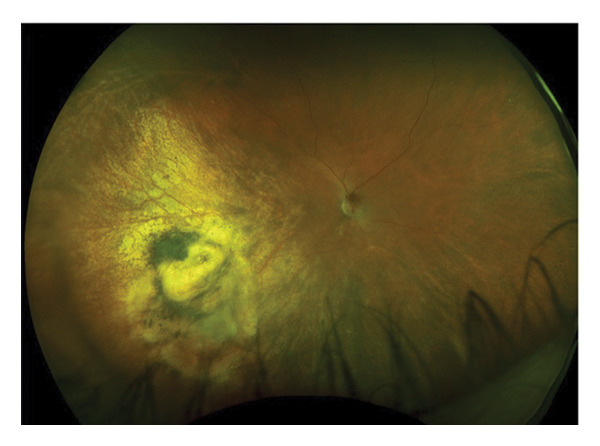
(e)

**Figure:**
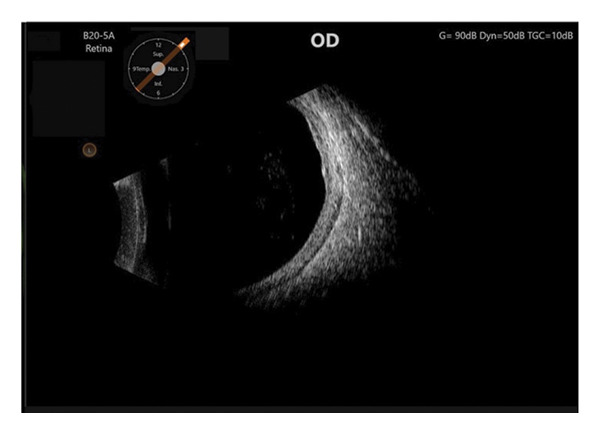
(f)

Preoperative systemic history was notable for a positive smoking history (quit 17 years earlier), hypertension that was well‐controlled on Hyzaar (losartan‐hydrochlorothiazide), and hyperlipidemia controlled with atorvastatin. The patient had no prior angina events or anesthesia complications. On the day of surgery, the patient was given 3 sets of topical dilating drops (1% tropicamide and 2.5% phenylephrine) 5 min apart without punctum manipulation or other method to enhance the penetration of the drops. Subsequently, a retrobulbar block of 4 mL of 0.75% bupivacaine and 1 mL of hyaluronidase was administered without complications. Surgery was initiated with a limited conjunctival peritomy and isolation of the superior and inferior rectus muscles. Lateral rectus was disinserted to allow plaque placement. Then, however, mydriasis was found to be insufficient for indirect ophthalmoscopy and FNAB. The eye was rotated for tumor localization when we observed that the pupil was inadequately dilated to view the fundus by indirect ophthalmoscopy, and the planned FNAB was performed. Three consecutive drops of presumed 2.5% phenylephrine were applied to the ocular surface to enhance mydriasis when the concentration was in fact 10%. Shortly thereafter, the patient complained of excess warmth and discomfort as his heart rate dropped to 30s, and the anesthesia team administered 0.2 mg glycopyrrolate. A subsequent change in his heart rate in the 120 s thus prompted the administration of 30‐mg esmolol. The patient’s blood pressure a few minutes later registered 215/134 mmHg and a heart rate of 161 bpm. The patient became agitated and attempted to pull off the sterile draping. Telemetry demonstrated sustained ST depressions consistent with demand ischemia caused by profound hypertension and supply demand mismatch. Over the next 30 min, both the patient’s heart rate decreased from 161 down to 78 bpm as well as his blood pressure from 215/134 mmHg to 95/52 mmHg, thereby indicating that the effects of the phenylephrine had subsided. General anesthesia was then induced with propofol, and an LMA was placed to successfully complete the surgical case.

During the postoperative monitoring period, the patient denied chest pain or shortness of breath. Blood pressure and EKG were normal. Out of precaution, serum troponin using a standard assay was found to be elevated at 0.15 ng/mL (normal levels: 0−0.04 ng/mL). The anesthesia team concluded that this finding was attributed to the acute cardiac stress experienced during surgery and not reflective of continued cardiac strain. The patient was kept overnight for observation. The following morning, a repeat troponin assay was unchanged (0.15 ng/mL), prompting the transfer of the patient to a formal inpatient systemic workup and heparinization in preparation for cardiac catheterization. Cardiac catheterization was found to be normal, thus demonstrating no evidence of obstructive coronary disease. Echocardiography and serum laboratory testing were unremarkable, other than a hemoglobin A1c of 5.9% (prediabetic). Troponin was down‐trending at 0.11 ng/mL, and cardiology considered the originally elevated troponin to be a spillage from ischemic myocardial injury during surgery. He was then discharged and followed as an outpatient.

Four days later, the patient underwent uneventful plaque removal as well as pupillary dilation. Twenty‐four months following plaque removal, the patient is doing well, with no other cardiovascular issues. He is monitored by his PCP periodically. His tumor has regressed successfully, and the exudative retinal detachment resolved (Figures 1(c) and 1(d)). Subsequent follow‐up of 2 years revealed stable tumor regression and no evidence of recurrence (Figures 1(e) and 1(f)).

## 3. Discussion

The use of topical medications for pupil dilation is critical to many surgeries performed by ophthalmologists [1]. Typical concentrations for tropicamide (anticholinergic) and phenylephrine (sympathomimetic) are 1% and 2.5%, respectively, administered in three rounds with each round separated by five minutes. For patients with darker and nonresponsive irides, ophthalmologists often provide additional rounds of the medications or utilize higher concentrations of these drugs.

Phenylephrine is a direct agonist of alpha‐adrenergic receptors and manifests not only with activation of pupillary dilation muscles but also with constriction of conjunctival arterioles and capillaries [3]. Matsumoto and colleagues demonstrated that the minimum effective concentration of phenylephrine‐induced mydriasis was 0.07% [4]. Animal testing by Chien and Schoenwald showed excellent bioavailability using 1% and 10% solutions, although the effect was not proportional to the concentration used [5]. In most clinical settings, 2.5% and 10% solutions are the predominant stocked formulations, with 2.5% being used most often and resulting in effective pupil dilation [1].

While generally safe, phenylephrine does harbor significant side effects if enough medication enters the systemic circulation. The principal route this drug enters the systemic circulation is through the nasolacrimal duct, which provides a larger mucosal surface area for the excess medication to be absorbed [6]. It has been estimated that up to 80% of the total administered eye drop volume enters systemic circulation through this route [7]. When enough phenylephrine enters the bloodstream, patients can experience minor side effects such as headache, sweating, and tremors. However, the three more severe side effects reported include hypertensive episodes, cardiac arrhythmias, and myocardial infarction (the former being the most prevalent) [6].

In 1988, Chua and Benrimoj compiled all studies investigating the effect of ophthalmic phenylephrine on systemic blood pressure and found 4 four studies associating the increased blood pressure with these drops and 8 denying such association [8]. The authors concluded that these mixed findings were due to diverse clinical factors (including patient age and past medical history) as well as variation of drug formulation. Other authors have proposed similar hypotheses [3, 9, 10]. Several more studies have shown similar results, with some cohorts reporting significant blood pressure elevation [11, 12], while others sustained no changes in blood pressure or heart rate [13].

More recently, a 2015 meta‐analysis collected 70 articles published between 1970 and 2014 and concluded that 2.5% phenylephrine drops did not significantly increase blood pressure or heart rate, but the 10% solution caused an average increase of 15 mmHg in blood pressure and 4.48 beats/min in heart rate shortly after administration that lasted 30 min on average. In 2022, Lodhi and colleagues conducted a prospective study on 517 patients undergoing cataract surgery using 0.8% tropicamide and 5% phenylephrine combination drop to investigate any adverse cardiovascular effects [14]. They reported a small increase in systolic blood pressure (5 mmHg) about 3 h after administration and did show an acute increase in heart rate five minutes after drops were administered attributing it to anxiety associated with surgery.

There were notable aspects in our patient’s ocular history, as well as how and when topical phenylephrine was administered, that highlight the risk of excess systemic absorption of this medication. First, as reviewed previously, topical 10% phenylephrine can have more pronounced effects on ocular vascular physiology, which by extension can affect systemic vascular physiology with equal magnitude. This was exquisitely illustrated by the patient’s ST depression and elevated cardiac troponins, indicating that the patient experienced an ischemic cardiac injury (even if transitory), which has been reported in the literature [15]. This patient would likely have performed well with limited additional 2.5% phenylephrine drops, and it is not uncommon that additional rounds of dilation using 2.5% phenylephrine is utilized in both the clinical and surgical settings. Second, the phenylephrine drops were administered after an inferior conjunctival peritomy. The tear film and conjunctiva are known barriers to systemic drug penetration, thus removing these barriers provided an easier means for systemic absorption of phenylephrine. None of the studies mentioned accounted for this scenario. Third, the presence of a vascularized choroidal melanoma may have altered the density of episcleral blood vessels and blood flow around the eye, perhaps accelerating systemic absorption of phenylephrine. We acknowledge this is difficult to prove yet worthy of mention.

Of note, this patient was transitioned to an acute coronary protocol that should not be deemed insignificant in any clinical or surgical setting. Although the cardiac catheterization reported no obstructive disease, the heart rate, blood pressure, and EKG readings during and after the surgical procedure characterized the patient’s cardiovascular status as suboptimal with demand ischemia. The anesthesia team was quick to recognize this and stabilized many of these metrics, but the consistently elevated troponin levels into the following morning prompted the team to activate the acute coronary protocol (i.e., heparinization followed by cardiac catheterization) and transferring the patient to the affiliated main university hospital. The engagement of all these resources in the context of routine outpatient eye surgery is rare, even in the context of patients who have complex medical conditions including heart failure, liver failure, or renal failure. However, the ability for the entire medical team to be vigilant in monitoring the vitals of patients during surgery is paramount, and the presence of a well‐trained anesthesia team to intervene quickly makes a significant difference in expediting high acuity care in situations such as the one illustrated in our case.

## 4. Conclusion

The application of topical ophthalmic phenylephrine should be performed with caution, particularly in situations where normal ocular barriers (e.g., tear film and conjunctiva) are disrupted. In these cases, fewer drops and lower dose of phenylephrine should be used in combination with copious rinsing of the ocular surface to remove excess medication after a short incubation period. In addition, the use of intracameral Shugarcaine or similar agents to achieve operative mydriasis should also be considered and reviewed with anesthesia. Finally, clear communication and providing monitored anesthesia care during locoregional anesthesia are key to stabilize the patient when excessive systemic absorption of phenylephrine occurs.

## Funding

This report has not received direct or indirect financial support.

## Consent

This study did not require ethics approval in accordance with local or national guidelines, as this is a single case report without identifying information.

## Conflicts of Interest

The authors declare no conflicts of interest.

## Data Availability

The data that support the findings of this study are available on request from the corresponding author. The data are not publicly available due to privacy or ethical restrictions.
